# A Rare Case of Hepatic Schwannoma in the Setting of Schwannomatosis

**DOI:** 10.7759/cureus.54705

**Published:** 2024-02-22

**Authors:** Minh-Anh Le, Rachel Shi, Justin Geraghty, Vania Zayat, Jignesh Parikh

**Affiliations:** 1 Internal Medicine, University of Central Florida College of Medicine/HCA Healthcare Graduate Medical Education, Orlando, USA; 2 Medical School, University of Central Florida College of Medicine, Orlando, USA; 3 Pathology, Orlando Veterans Affairs Medical Center, Orlando, USA; 4 Pathology, University of Central Florida College of Medicine, Orlando, USA

**Keywords:** spinal schwannoma, schwannoma, schwannomatosis, neurofibromatosis, hepatic schwannoma

## Abstract

Schwannomas are benign nerve sheath tumors commonly found in the head, neck, vestibular system, and extremities. Primary hepatic schwannomas are exceptionally rare, with 34 cases reported to date according to our review of the literature. This case report describes a 79-year-old man with a medical history of skin and thyroid cancer, who presented with no clinical symptoms and underwent a follow-up MRI due to an initial scan indicating a suspicious hepatic mass resembling an atypical hemangioma. The MRI revealed a 3.6 cm left hepatic mass concerning for an intrahepatic cholangiocarcinoma. Histopathological and immunohistochemical studies of a biopsy of the liver mass confirmed the presence of a benign hepatic schwannoma. Further evaluation revealed multiple spinal schwannomas, leading to the diagnosis of schwannomatosis. The diagnosis of hepatic schwannomas poses challenges through imaging alone. This case underscores the importance of microscopic evaluation in accurately diagnosing hepatic masses. Additionally, the presence of concurrent schwannomas should be considered in patients initially diagnosed with isolated schwannomas.

## Introduction

Schwannomas, previously or in other settings known as neurilemomas or neurinomas, are rare benign nerve sheath tumors derived from Schwann cells. These encapsulated tumors typically occur in motor or sensory nerves and are commonly found in the head, neck, vestibular system, extremities, or posterior mediastinum [1]. While the diagnosis spans ages 20-50 years and affects both genders, recent case reports suggest a higher incidence in females. Primary hepatic schwannomas are exceptionally rare, with fewer than 30 cases reported before 2016 [2]. We conducted a literature search in PubMed from 2016 to 2023, resulting in four additional cases of primary hepatic schwannomas. To our knowledge, concurrent occurrences of hepatic schwannomas with spinal schwannomas have yet to be reported.

## Case presentation

A 79-year-old Caucasian man with no presenting complaints underwent a one-year follow-up MRI after the previous scan showed a left hepatic lobe lesion favored to represent an atypical hemangioma. The patient had a past medical history of basal cell carcinoma of the right nasal ala that was removed with Mohs surgery and thyroid cancer that was treated with total thyroidectomy. His chronic conditions at the time of presentation included essential hypertension, chronic kidney disease, and chronic obstructive pulmonary disease. Abdominal examination and laboratory tests, including tumor markers (cancer antigen 19-9, carcinoembryonic antigen, alpha-fetoprotein) and liver function tests, were unremarkable.

**Figure 1:**
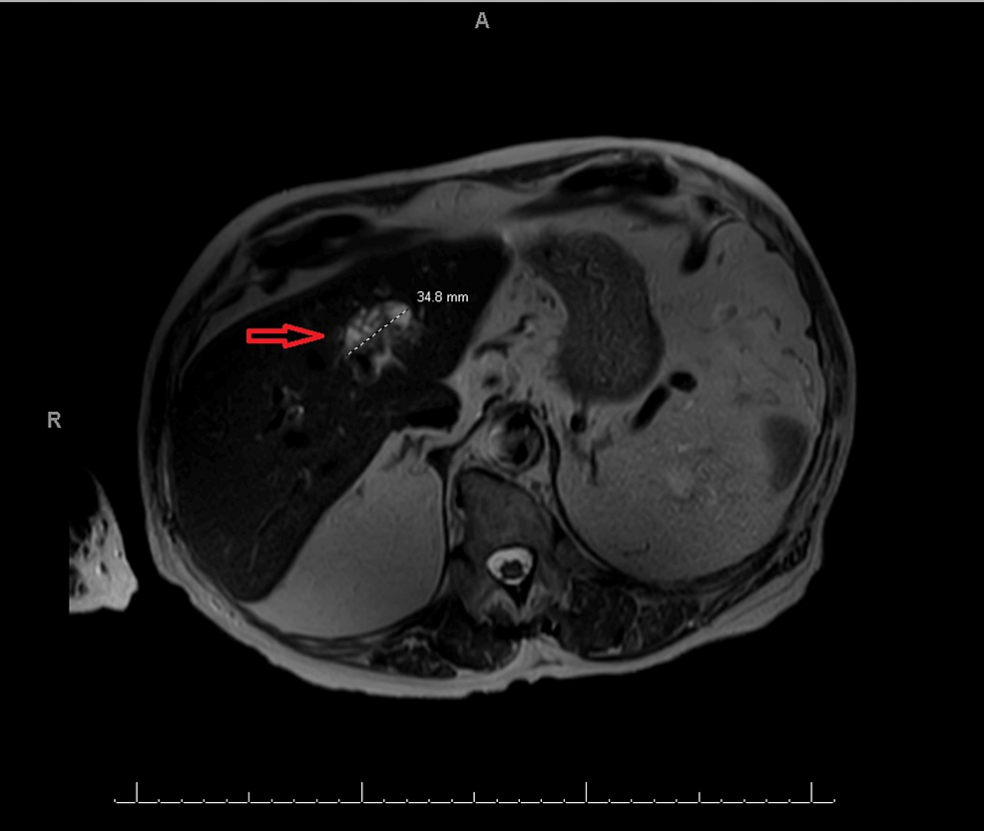
MRI of the abdomen showing an ovoid heterogeneous T2 hyperintense lesion (arrow) at the junctional segment II/IVa with mild extension into segment III/IVb with intrinsic heterogeneous and linear hypointense signal intensity.

The current MRI (Figure 1) showed a 3.6 cm mass in the left hepatic lobe that was suspicious of a neoplastic process, this time concerning for intrahepatic cholangiocarcinoma. It was described as an ovoid heterogeneous T2 hyperintense lesion at junctional segment II/IVa with mild extension into segment III/IVb with intrinsic heterogeneous and linear hypointense signal intensity. To further evaluate the mass, a fine-needle aspiration was performed, yielding hypocellular results with predominantly stromal cells. Subsequently, a core biopsy was performed due to the possibility of an unsampled lesion.

**Figure 2:**
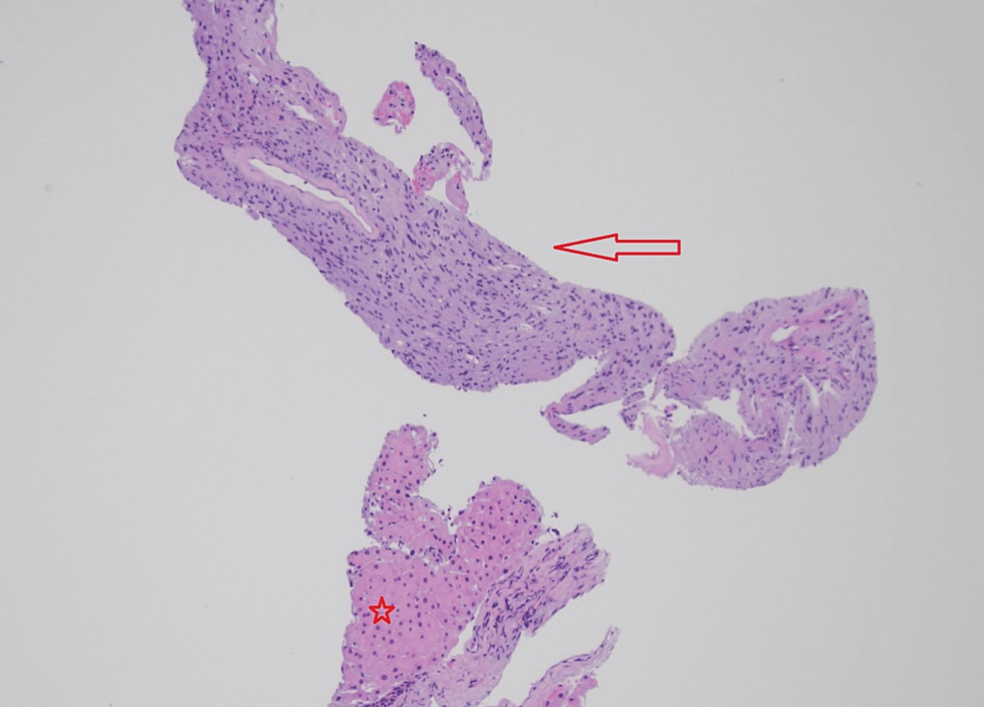
CT-guided needle core biopsy showing a cellular neoplasm (arrow); adjacent benign hepatic parenchyma is apparent (star) (hematoxylin and eosin, 10× magnification).

**Figure 3:**
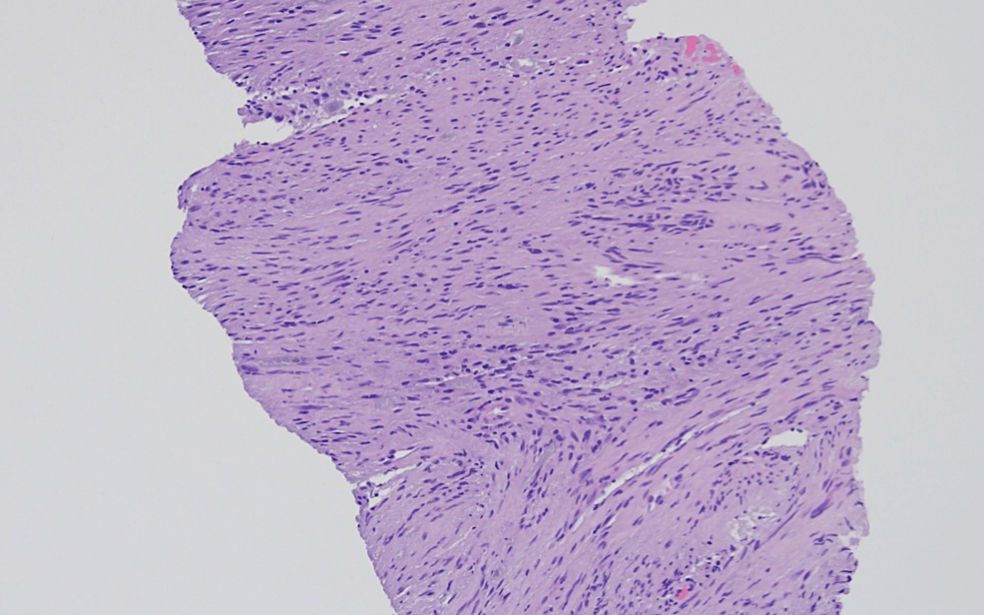
CT-guided needle core biopsy showing that the tumor is composed of spindle elongated cells with focal nuclear palisading (hematoxylin and eosin, 20× magnification).

**Figure 4:**
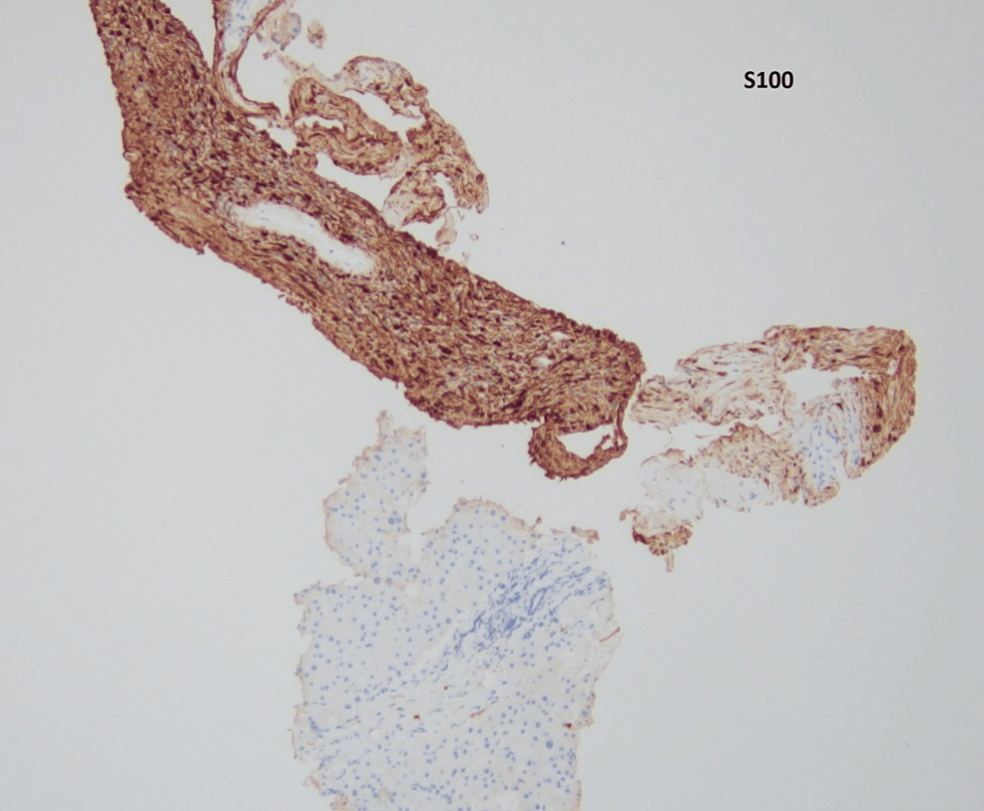
S100 stain of the liver biopsy at 10× magnification showing that tumor cells are positive for S100 (brown staining) and negative in the adjacent normal liver parenchyma.

**Figure 5:**
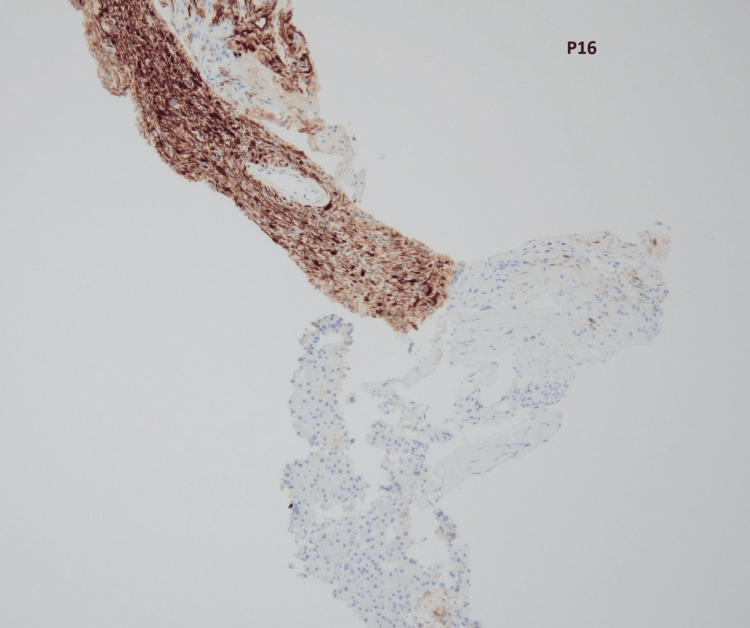
p16 stain of the liver biopsy at 10× magnification showing that tumor cells are positive for p16 (brown staining) and negative in the adjacent normal liver parenchyma.

The core biopsy (Figures 2, 3) demonstrated a cellular lesion composed mostly of elongated spindle cells (Antoni A areas) with focal vascular network without any atypia or an increase in mitotic activity compatible with a benign spindle cell neoplasm, including schwannoma, gastrointestinal stromal tumor, or leiomyoma, among others. The lesion showed positive staining for S-100 (Figure 4), p16 (Figure 5), and SOX10, while showing negative staining for MCK, CD34, CD117, desmin, SMM-HC, MELAN A, synaptophysin, calretinin, and CD31. The biopsy findings along with the immunohistochemical stain results were consistent with a schwannoma, clinically presumed to be an intrahepatic primary.

Additionally, the abdominal MRI showed an incidental finding of nonspecific focal enhancement within the L4/5 spinal canal. Therefore, a subsequent MRI of the lumbar spine was performed, showing multiple scattered small enhancing lesions within the thecal sac and a 1.7 cm intrathecal mass causing severe spinal canal stenosis at the L4-L5 level. The patient at that time had a complaint of lower back pain and lower extremity weakness, so he was referred to a neurosurgeon and underwent resection of the L4-L5 Intradural extramedullary spinal tumor. Pathological examination confirmed the mass as a schwannoma. With this result, a diagnosis of schwannomatosis-not otherwise specified (NOS) could be made without genetic testing, according to the most recent consensus in the diagnostic criteria of schwannomatosis published in 2022 [3].

The management for the hepatic schwannoma in this patient was nonsurgical according to a multidisciplinary healthcare tumor board composed of gastroenterology, radiation oncology, interventional radiology, and general surgery in our facility due to the lack of any clinical presentation and low risk of progression to malignancy of schwannomas in general. Clinical monitoring and repeating imaging for surveillance purposes are indicated. The patient and his family might benefit from genetic testing.

## Discussion

A schwannoma is a rare, benign tumor originating from Schwann cells which are peripheral nerve sheath cells. While schwannomas commonly occur in the head, neck, and upper extremities, they can rarely be found in the abdomen [4]. On imaging, schwannomas typically exhibit hypointense T1-weighted and hyperintense T2-weighted MRI findings, along with a heterogeneously enhanced margin [1,5]. Preoperative diagnosis of schwannomas can be challenging. Microscopic examination reveals highly ordered cellular regions known as Antoni A areas, characterized by interdigitating processes and nuclear palisades forming Verocay bodies. Additionally, Antoni B areas, which consist of loosely organized, myxoid regions with hyalinized blood vessels, degenerative changes, and thin wispy cells, can be observed [6].

The occurrence of intrahepatic schwannomas is exceedingly rare, with only approximately 34 reported cases in the medical literature. Schwannomas in the liver can originate from the splanchnic or vagal nerve systems that innervate the organ. Patients may present with epigastric tenderness, abdominal discomfort, and nausea or may remain asymptomatic. Due to their rarity and similarity to other neoplasms, preoperative diagnosis of liver schwannomas can be challenging. Radiologically, these tumors may resemble intrahepatic cholangiocarcinoma and gastrointestinal stromal tumors [7]. In certain cases, larger tumors can undergo secondary degeneration, hemorrhage, or calcification, leading to misdiagnosis as liver metastases or degenerative hydatid cysts [2,6]. Moreover, the presence of vague gastrointestinal symptoms or asymptomatic presentation further complicates accurate diagnosis. A fluorodeoxyglucose-positron emission tomography typically shows high metabolism indicating possible malignancy; therefore, it does not aid with the diagnosis of schwannomas [8,9].

Schwannomas are well-delineated nerve sheath tumors that are almost always benign (>95%). Malignant peripheral nerve sheath tumors are extremely rare, affecting about 1.46 out of every 10 million people each year [10]. They may arise from a peripheral nerve or a pre-existing neurofibroma; in about 50% of cases, these tumors arise in the context of neurofibromatosis type 1. Radiation is also a risk factor for malignant schwannomas and it portends a poor prognosis [11]. Histopathology demonstrates infiltrative growth, hypercellularity, cytologic atypia, occasional mitotic figures, and tumor necrosis [12]. Differentiating between benign and malignant hepatic neoplasms is challenging as CT and MRI do not easily distinguish between the two [5]. Malignant schwannomas have a poor prognosis, making surgical resection crucial for a definitive pathology diagnosis, with complete excision often being curative [2,5]. In this case, the patient presented with vague symptoms and an inconclusive MRI and fine needle aspiration. Only after performing a CT-guided needle core biopsy was performed, the proper diagnosis was made. The management of benign schwannomas largely depends on the tumor’s location and the presence of clinical symptoms. It may involve either a watch-and-wait approach (as in the case of this patient, who did not exhibit abdominal symptoms) or surgical resection. Follow-up recommendations for benign schwannomas typically involve a contrast-enhanced CT scan every three months for the first two years, followed by imaging every six months for the next two to five years, and annual imaging thereafter [2]. Benign schwannomas generally have favorable outcomes, with a low risk of relapse [2].

The presence of coexisting spinal schwannomas with the hepatic schwannoma in this patient suggests the possibility of an underlying genetic disorder. Schwannomatosis is characterized by a predisposition to form benign nerve sheath tumors, in the absence of intradermal and bilateral vestibular schwannomas. In 2022, the nomenclature for neurofibromatosis type 2 and schwannomatosis was further updated to recognize the disorders as part of a spectrum of schwannoma predisposition syndromes with extensive clinical overlap [3]. If genetic testing is not performed or is not available (as is the case for this patient), a diagnosis of schwannomatosis-not otherwise specified) can be established having met the following two criteria: (1) imaging evidence of multiple non-intradermal schwannomas and (2) histopathological confirmation of at least one schwannoma or hybrid nerve sheath tumor. If genetic testing is available, the diagnosis of schwannomatosis can be further classified into neurofibromatosis type 2-related schwannomatosis, *SMARCB1*-related schwannomatosis, *LZTR1*-related schwannomatosis, or 22q-related schwannomatosis [3].

## Conclusions

Liver schwannoma is a rare tumor arising from peripheral nerve sheaths. When a liver mass exhibits unclear characteristics on imaging, a biopsy is essential for an accurate diagnosis. Due to the rarity of this condition and the broad range of liver lesions, misdiagnosis can occur without microscopic evaluation. It is particularly crucial to consider schwannoma as a differential diagnosis in patients with predisposing risk factors such as neurofibromatosis or concurrent schwannomas.
